# The Lebanese Regie state-owned tobacco monopoly: lessons to inform monopoly-focused endgame strategies

**DOI:** 10.1186/s12889-022-13531-z

**Published:** 2022-08-29

**Authors:** Hala Alaouie, J Robert Branston, Michael John Bloomfield

**Affiliations:** 1 Department of Social and Policy Sciences, University of Bath, Bath, UK; 2 Department for Health, Tobacco Control Research Group, University of Bath, Bath, UK; 3 School of Management, University of Bath, Bath, UK

**Keywords:** Tobacco industry, Tobacco endgame, Monopoly, State-owned tobacco monopoly, Tobacco control, Tobacco policies

## Abstract

**Background:**

Many countries have started pursuing tobacco ‘endgame’ goals of creating a ‘tobacco-free’ country by a certain date. Researchers have presented models to attain this goal, including shifting the supply of tobacco to a monopoly-oriented endgame model (MOEM), wherein a state-owned entity controls the supply and distribution of tobacco products. Although not designed to end tobacco use, the *Regie* in Lebanon exhibits some of the key features identified in MOEM and hence can serve as a practical example from which to draw lessons.

**Methods:**

We comprehensively review previous literature exploring tobacco endgame proposals featuring a MOEM. We distil these propositions into core themes shared between them to guide a deductive analysis of the operations and actions of the *Regie* to investigate how it aligns (or does not) with the features of the MOEM.

**Results:**

Analysing the endgame proposals featuring MOEM, we generated two main themes: the governance of the organisation; and its operational remit. In line with these themes, the investigation of the *Regie* led to several reflections on the endgame literature itself, including that it: (i) does not seem to fully appreciate the extent to which the MOEM could end up acting like Transnational Tobacco Companies (TTC); (ii) has only vaguely addressed the implications of political context; and (iii) does not address tobacco growing despite it being an important element of the supply chain.

**Conclusion:**

The implementation of tobacco endgame strategies of any type is now closer than ever. Using the *Regie* as a practical example allows us to effectively revisit both the potential and the pitfalls of endgame strategies aiming to introduce some form of monopoly and requires a focus on: (i) establishing appropriate governance structures for the organisation; and (ii) adjusting the financial incentives to supress any motivation for the organisation to expand its tobacco market.

## Background

Eight countries (Australia, Canada, England, Finland, Ireland, Malaysia, New Zealand, and Scotland) have articulated specific endgame goals as part of their drive to become ‘tobacco-free’ [1–5]. Most of those countries intend to decrease tobacco use and reduce smoking prevalence to below 5% of their population by a predetermined date [2, 6]. While there is no consensus on how to reach these goals [4], scholars have suggested a wide range of possible models to achieve endgame strategies [7–17]. Among the suggested proposals is the creation of monopoly oriented endgame models (MOEM). These feature organisations that have public health mandates to control one or more aspects of the tobacco supply chain, and which exhibit monopoly like features [8, 9, 11, 17–21]. Essentially, this means that production of, and trade in, tobacco products is undertaken in the public interest through a state-run entity.

These models remain largely theoretical as none have yet been applied in practice, so there are many uncertainties about their potential implications [1, 22], which therefore makes it difficult to evaluate them [6, 23, 24]. Consequently there is value in comparing the proposed MOEM to an existing context [3] to be able to assess their potential applicability in real-world settings and accordingly draw lessons for advancing endgame strategies.

While certainly not the same, there are a few state-owned tobacco monopolies (SOTM) that do exist in various markets, which are similar in a number of ways to MOEM because of the monopoly characteristic. The market dynamics between the two organisational structures will be different (especially in regard to tobacco control policies) given that the monopolies exist for different reasons, but their nature as monopolies nevertheless offers a potentially useful point of comparison. As such, it should also be noted that there are a number of other state-owned (or part state-owned) tobacco firms that have less than 100% of the market (see [22, 25]). However, the oligopolistic nature of such firms suggests that the associated market dynamics will be further removed from those of an MOEM and hence they are not the focus of the study herein.

**Fig. 1:**
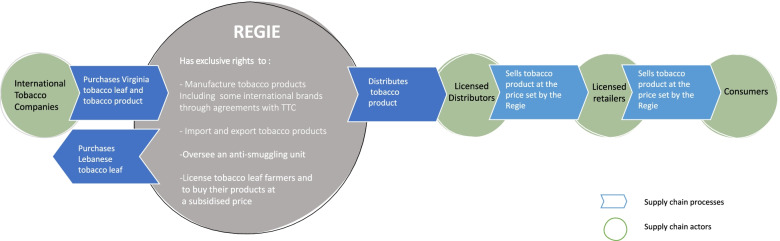
The case of the *Regie*: elements of its tobacco supply chain. Source: Authors’ interpretation, drawing upon [28, 29, 31]

Lebanon, despite the on-going economic crisis, is an upper middle-income country (UMC) [26], and is one of the few countries with an operating SOTM, the *Regie Libanaise de Tabacs et Tombacs*, or the *Regie*, as it is commonly called. In this paper we will explore the *Regie*, as an exemplar SOTM. Within Lebanon, the tobacco market is controlled by the *Regie,* which is a commercial public institution [27, 28] with exclusive rights to undertake: domestic leaf purchasing of locally grown tobacco; manufacturing and distribution of tobacco products in Lebanon, including export/import activities [29]; and overseeing an anti-smuggling unit [30] (See Fig. 1 for a visual overview of the Regie).

While endgame strategies seem far from the agenda of the Lebanese Government [2], as the *Regie* is not designed to reduce tobacco use but rather to derive revenue for the government from its manufacture/sale, it exhibits a significant number of the key features that have been identified in MOEM. Thus, the *Regie* can serve as a practical case to explore some of the opportunities and challenges one could expect if an MOEM was to be adopted.

This paper therefore aims to investigate the extent to which the *Regie*, as a SOTM, aligns (or does not) with ideas around suggested MOEM. We map the features of the suggested theoretical endgame proposed models featuring MOEM into core themes shared between them. This then guides an exploration of the *Regie’s* structure, operations, and action relative to these themes in order to draw lessons from the *Regie* for advancing endgame strategies in a real-world setting and in doing so, also consider lessons for advancing tobacco control in Lebanon. Furthermore, this paper also contributes to the existing literature on state-owned tobacco monopolies, given we are the first to investigate the *Regie* or any state-owned tobacco monopoly in the context of tobacco endgame strategies.

## Methods

To identify endgame literature suggesting MOEM, firstly, we began by reviewing the special issue of Tobacco Control on endgame strategies published in May 2013 [7] and drew on a paper by McDaniel et al. [6], containing a comprehensive list of all endgame proposals up to 2016. Secondly, for possible proposals published from 2016, we searched the PubMed database and Google Scholar using the terms ‘(tobacco AND endgame)’ AND (Monopol* OR state-own* OR state-run* OR state-govern*) and then conducted a ‘snowball’ search to find further relevant articles from those previously identified. The result of the search yielded a total of 38 papers after duplicates were removed. We excluded all endgame proposals that did not propose utilising an MOEM responsible for manufacturing and/or marketing and/or retailing. Overall, we identified a total of 7 proposals [8, 9, 11, 17, 18, 20, 21] that suggest a role for state-owned models of tobacco supply as part of an endgame strategy (Table 1).

**Table 1:** The characteristics and different features of endgame proposals that support introducing monopoly-oriented endgame models (MOEM)

Study	Aim	Proposed MOEM	MOEM function within the supply chain
Liberman [17]	• Tackle the “perverse incentive” of tobacco companies to break the nexus between profit making and harm causing via a regulatory system • To minimise harm caused by tobacco	An agency would oversee the whole regulatory system and fill in ‘regulatory gaps’ within the supply chain	The agency has an overarching regulatory presence on all the processes of the supply chain
Borland [8]	• Regulate the tobacco industry, by incentivising them to produce less harmful product via regulating the market • To minimize the harm of tobacco product	Tobacco product agency (TPA) would act as a health-oriented intermediary that would remove the marketing power of the tobacco industry from the supply chain	Act as an intermediary between the manufacturers and the distributors: take over the marketing component
Callard [9]	• Transfer the responsibility of tobacco product manufacturing from a profit making to a non-profit making institution via taking over the work of the tobacco industry and achieve health goals • To reduce tobacco, use in a timely manner	A non-profit agency (NPE) with public health mandate to take over the work of the tobacco industry	Transfer the supply chain work to a non-profit agency removing the tobacco industry from the whole market
Thomson et al. [18]	• Provide New Zealand government with a proposal on how to regulate the tobacco market • To maximize harm reduction and eliminate marketing of branded tobacco	Establish a government tobacco authority monopoly (to use an approach like the one suggested by Borland)	Similar to Borland but also proposes regulating the retailers by binding them to a license agreement
Thomson el al [11]	• Eliminate the availability of commercial smoked tobacco within a 10-year period to reach ‘(near) zero’ sales • To be able to ensure the best health and social outcomes for people	A government agency that would run a ‘sinking lid’ quota on tobacco product to be auctioned to manufacturers and importers. The manufacturer can only sell the traded amount to distributors	The agency will be introduced before the manufacture in the supply chain and will provide suppliers with a tradable quota
Gray [19, 20]	• Regulate the toxicity of tobacco products and entirely transition to less harmful products within a 5 year period • To shift consumers towards less harmful product	Suggests a government run tobacco marketing monopoly (to use an approach like the one suggested by Borland)	Same as Borland
Smith et al. [21]	• Introduce the concept of moving tobacco sales to government owned outlets as a progressive step towards the tobacco endgame	A government tobacco monopoly to regulate the pre-existing “government-operated alcohol retail monopolies”- to start selling tobacco product	A government tobacco monopoly be introduced to act as sole buyer from the manufacturers and remove the retailer from the supply chain and replace them by government operated retailers

Our study did not aim to undertake an in-depth analysis of the endgame proposals or to evaluate them, as other papers in the literature have done so [1–3, 6, 10, 15, 32–37]. Instead, we aimed to map the different features of the proposals to understand how the authors are proposing to integrate MOEM to advance endgame strategies. We therefore adopted a qualitative thematic analysis using a codebook approach [38, 39] which consists of systemically analysing the endgame proposals to provide a framework for analysing the *Regie*. Accordingly, we identified two main themes and nine subthemes from the endgame proposals. The generated themes were used deductively to conduct a document analysis (a systematic way to examine and synthetise data collected from documents [40]) to analyse the *Regie* and investigate how it aligns (or does not) with the features identified as being required for advancing the tobacco endgame. All three authors were involved in the exploration of the endgame literature and coding around them. These codes were discussed, refined, debated, and agreed by all three authors who collectively generated the final themes and sub-themes**.**

The first author collected and reviewed the wider literature on the *Regie* and undertook the primary data collection, which was then discussed extensively with the other two authors. The primary data on the *Regie* was collected by searching its website (https://www.rltt.com.lb). Given the nature and the source of these documents, they were triangulated [41] whenever possible with other document sources such as multiple government departments’ documents and websites [29, 42], the Global Tobacco Industry Interference Index (GTIII) 2020 [43], existing academic literature covering the *Regie* and/or tobacco control in Lebanon [27, 28, 31, 44–54], and mainstream Lebanese newspapers and online news platforms (such as The Daily Star, Al Akhbar, An Nahar, Al Modon, Al Janoubia). To locate news articles addressing the *Regie*, a search of the website of those newspapers/online news platforms was conducted via a Google search in both Arabic and English using the *Regie* as a key word. Inclusion criteria consisted of any articles addressing the two identified themes. The earliest media article identified, extracted, and found to be relevant date back to 2007 and the search extended until early September 2021 [55].

## Results

Of the seven identified proposals to suggest the use of an MOEM, the majority [8, 11, 17, 18, 20, 21] suggested regulating the tobacco industry while only one proposal [9] aimed to take over the tobacco industry. In the following sections, we offer an overarching synthesis of the main features suggested by the endgame proposals by theme and then sub-theme. We then compare each theme to data from documents identified on the operation of the *Regie* to critically analyse the extent to which the *Regie’s* features align with the relevant ideas around MOEM.

### Governance structure

Our analysis consisted of identifying data from the seven proposed endgame proposals with respect to the first theme, governance structure, which we summarise in Table 2 according to the sub-themes of: ownership and control, reporting, and financing. The findings from Table 2 will be referred to in each of the next three sections when comparing the governance of the *Regie* to the endgames proposals.

**Table 2:** Summary of the characteristics of the governance structure theme of proposed monopoly-oriented endgame models (MOEM)

Sub-themes	Liberman [17]	Borland [8]	Callard [9]	Thomson et al. [18]	Thomson et al. [11]	Gray [19, 20]	Smith et al. [21﻿]
Ownership of the MOEM							
(1) Government (2) Ministry of Health	The ownership was not specified but it need to be distant from executive governmental body	(1-Or equivalent that is different from the department that collects taxes that could be the department of finance or equivalent)	Ownership was not discussed. But it was stated it could be public or private. It could be similar to pre-existing models: water utilities, public broadcasting, hospital systems	(2)	(1)	(1)	(1)
Control—External							
(1) Established by legislation to act under charter and certain criteria (2) To have PH mandates/goals (3) To ensure control of illicit trade (4) To report on its work to an independent body (5) To require the TTC to disclose information on its research and products (6) To have the power to set differential tax in co-ordination with treasury	(1)(2)(3) (4- To report to a federal/state body in the same field of work such as drugs, health.) (5- The MOEM will incentivise tobacco manufacturers towards developing less harmful/addictive products)	(1)(2)(3) (4) (5- The MOEM will incentivise them to disclose information to the TPA) (6)	(1) (2)	(1)(2)(4) (5- Disclosure will be to the Tobacco Authority or Ministry of Health)	(1)(2)(4- Reporting will aim at evaluating the phasing out) (5-The MOEM will aim to implement Article 5.3)	(1)(2)	(1)(2)(3)
Control—Internal							
(1) Establish a board of experts from different fields (2) To have board members distant from executive government and political interference to ensure independence	(1)(2)	(1)(2)	Unspecified	(2- Just mentions independent)	Unspecified	Unspecified	Unspecified
Reporting mechanism							
The MOEM transparently communicates information by making it publicly available This information includes: (1) Meetings (2) Decisions (3) Operations (4) Price changes (5) About products (6) Licensing (e.g., retailers or farmers) (7) Deals/agreements with TTC	(1)(2)(3)(5)(6)(7) Reporting mechanism should be made possible (a) By routine publication on the internet (b) Given the freedom of information legislation	(2)(3)(4)(5)(7)	Unspecified	Unspecified	(4)	Unspecified	(4)(5)
Financing- Source to cover cost of operation							
(1) Government budget (2) Self-financed through business (3) From tax revenue	Unspecified	(1)(2)	(2)(3)	(3)	(2- Revenue from quota bidding on residual tobacco) (3)	(3)	(2)
Financing- means to address financial interest / vested interest /corruption							
(1) Earmark the profit for tobacco control and CSR activities to avoid reliance on tobacco sales (2) Gradually drop tax revenue in line with decrease in tobacco consumption to avoid the government incentive to maintain sale (3) Incentivising staff to maintain the agency integrity/involve them in cessation programmes (4) Hold staff accountable for their work (5) Benchmark the work of the agency toward another agency in jurisdiction (6) Isolate the MOEM decision making regarding revenue (7) Decrease dependence on revenue by setting a date to eliminate tobacco	(6)	(3)(6)	(2)	Unspecified	(1)	(1- Fund anti-smoking activities) (4)	(1- Provide retailors with one-time transition fund to shift to other business; for education; enforcement of the ban.) (7)

### Ownership and control

The endgame literature suggests that the proposed MOEM should be under the ownership of the government. The board of directors should contain ‘expert members’ to remain distant from government and political interference, thereby ensuring independence. It did not address how board members or staff are appointed to ensure independence, but it was mentioned [8] there is a need to account for staff integrity. The endgame literature also proposed that the decisions of the MOEM and the TTC need to be monitored by an independent body.

Comparing the *Regie* to those features, the *Regie* is owned by the Lebanese government and under the jurisdiction of the Ministry of Finance (MOF) [30] which oversees its technical, economic, and financial management. By law [29, 56] the *Regie* is supervised by an appointed government commissioner and subject to financial audit by the MOF. The government commissioner is the communication liaison between the *Regie* and different governmental institutions. The *Regie* is only supposed to directly interact with the MOF twice per year regarding its financial budget [29]. Technically, this means the *Regie* is expected to maintain distance from the executive government and avoid political interference, an aspect that is broadly in line with the endgame ideas.

In practice, however, this is not the case. Firstly, the board is not as independent as it is supposed to be given the Lebanese political system is based on sectarianism that balances power between the different religions/sects that exist [28, 31, 57]. Hence, within this system, the *Regie* is under the patronage of the head of the parliament [28] where the general manager, in place since 1993 and appointed as chairman of the administration committee since 2002, is affiliated to the political party of the head of parliament. In addition, since 2016, the MOF has been run by to the same political party [58]. Furthermore, within this sectarian system almost half of all *Regie* employees are not hired based on merit but simply on the nature of their affiliation, leading to a clientelism relationship (i.e. creating a system of favours and associated loyalty) [28, 31, 59, 60]. Secondly, there are no mechanisms in place to control and hold the *Regie* and TTC accountable for their actions [61]. Hence holding its employees accountable is difficult as well [62]. Lebanon, a signatory of the WHO Framework Convention on Tobacco Control (FCTC) (an international treaty which aims to protect people from the harm of tobacco at social, environmental, economic, and health levels) [63] since 2005 [64], is expected to have its National Tobacco Control Programme (NTCP) [65] within the Ministry of Public Health to monitor and evaluate tobacco industry work (including the *Regie*) in line with the provisions of Article 5.3 of the FCTC [66] (Guideline 8 in Article 5.3 recommends that SOTM should be treated the same as the tobacco industry [67]). However, since at least early in 2019 and likely considerably earlier, the NTCP is practically inactive, claiming a lack of funds [64]. Furthermore, according to the law, the state commissioner has the right to monitor the *Regie’s* work and decisions, and even summon and interrogate high-ranking employees. However, if/how they actually monitor the *Regie* is not clear, with significant corruption and bribery having been reported at the *Regie* [59]. Indeed, both the state commissioner and the financial auditor’s salaries are paid by the *Regie* [68], which raises questions with respect to independence.

In the absence of independence, and with close link to a political party, the *Regie* is not distant from political interference. The GTIII 2020 ranked Lebanon as the fourth worst country on the overall country ranking (with the score deteriorating 1 point from the 2019 report). It scored poorly for: governmental institution and/or officials’ interaction with the tobacco industry (including the *Regie*) [43]. To illustrate, in 2011 Lebanon passed its first comprehensive tobacco control law [42], which includes imposing regulatory measures on the tobacco industry, and hence the *Regie*. The tobacco control laws in Lebanon (like other laws) need to be signed-off by more than one ministry, giving each a power of veto. Any tobacco control measures that restrict the operations of the *Regie* will likely damage its profitability, and hence (negatively) influence the decision of the MOF [53]. See for example, the same for pictorial warning as discussed in the product design section below.

### Reporting mechanism

The endgame literature suggests that the proposed MOEM should make its information (meetings, discussions, decisions related to addressing health outcomes, licensing arrangements, price changes, etc.) publicly available through different platforms, as well as/or report to an independent overseeing body to ensure transparency.

Comparing the *Regie* to the endgame models’ suggestions, the *Regie* does report on its activities, but in a very similar way to a TTC, who typically produce yearly annual reports and publish on their websites that give an overview of select activities (but with few substantive details). While the *Regie* does not produce such an annual report, it does have a website (https://www.rltt.com.lb/), which includes similar information. One notable difference is that the website also includes information about the brand-level retail prices set for all shops in Lebanon, which are updated on a regular basis in line with the set market price. However, it seems that the published information on price is more about controlling the market rather than about transparency in its activities, as wholesalers and retailers are required to sell at the recommended retail price. A requirement not always followed (see below section on wholesalers).

Crucially though, the *Regie’s* financial disclosures are weak. It does not publish information on: the costs of manufacturing local brands; the cost of packaging; the salaries of its employees [46]; the special deals it gets on the price of imported tobacco products from the TTC (thought to be a lower price than other countries in the region) but kept secret at the request of the TTC [54]; the profit margins it earns on tobacco sales of domestic brands; or the profits made on the domestic production of international brands.

### Financing

The endgame literature suggests the MOEM should or can be a non-profit institution and proposes how the MOEM could be financed. The different proposals raise concerns with respect to the MOEM’s financial interest in the sale of tobacco that could lead to corruption and/or dependence on revenues. Hence, they propose mechanisms on how to address potential dependence of governments on tobacco revenues.

Unlike the suggested endgame proposals, the *Regie* is a profit seeking organisation with ‘net profits, losses, and expenses’ directly linked to the public treasury [49]. It aims to expand the Lebanese tobacco market, thus its revenues and profits [30, 69, 70]. As per its general manager/chairman, their aim is to “*win (a) place among internationally sustainable pioneer tobacco companies”* [71, 72]. Part of the government tax revenue collected from imported tobacco products is used to cover the government Price Support Program (PSP) where the *Regie* purchases tobacco leaf from tobacco farmers at a given yearly price and specified quantity set by the MOF [49].

As stated above, the endgame proposals raise concerns with respect to financial interests, and an examination of the *Regie* confirms the need to be concerned. In 2019, the MOF praised the *Regie* for its 34% revenue increase relative to 2018 [73]. A news article pointed to the occurrence of high levels of corruption within the *Regie* [74] and another reported bribery of the financial audit of the *Regie* by the MOF to cover-up the illegal activities of the *Regie* [59]. Furthermore, a 2009 article reported that the *Regie* was, as of 2009 (no current data as to whether this procedure still in place), consulting retired employees who received compensation of up to LBP7 million per month per person (then US$4600 as this paper did not account for the financial crisis that Lebanon is currently experiencing since October 2019; all prices are based on the fixed exchange rate pre-October 2019, of LBP1507.5/US$1). The article added that one of those consultants, a former senior employee, was arrested at the behest of the *Regie* for vested interest related matters.

Also of concern are the financial interests of some of the political elites given their vested interest in the tobacco industry [75–78]. The TTC bypass the *Regie* by directly lobbying politicians with respect to tobacco laws [51]. Indeed some member of parliament (MPs) are in close connection with tobacco traders [75], have a stake in the main hospitality sectors in Lebanon and therefore they have an interest in amending the tobacco control law for their financial gain [79, 80]. Hence, some MPs have suggested amending tobacco control legislation to be more lenient on the hospitality sector that offer waterpipe indoors, claiming that the tobacco control law is negatively affecting tourism-related revenue. Similarly, a tobacco advocate reported that MPs have vested interests in preventing tax increase, which could explain why attempts over six years to raise tobacco taxation have always been blocked by self-serving MPs [51, 75].

In addition, the “citizen budget” [81], a publicly available report prepared by the MOF, includes data on the ‘total tax revenue’ from the *Regie.* By comparing the information on the *Regie* website in 2020 (net profit LBP268bn) and the data from the citizen budget report (*Regie* profits of LBP200.2bn) [73], the numbers do not match. A discrepancy that is not reported by or explained in the financial audit of the Regie by the MOF.

The political context is also a barrier towards regulating the *Regie*’s finances. Back in 1991, a committee to operate the *Regie* was established within it*,* without any legal standing or clear job description and on a temporary basis, but it is still in operation today without clear benefit for the *Regie* administration. A former Minister of Finance reported that it has been costing the state around LBP1bn (US$666.6 million) per year [82]. Despite his request and subsequent requests by other Ministers of Finance to abolish it, the Prime Minister in office has declined under the pretext of having to preserve the political balance sharing system within the public administration [82].

### The supply chain operational remit

We also analysed data from the seven endgame proposals with respect to the second theme, operational remit, which are outlined in Table 3 according to the sub-themes of: product design; purchase; promotion; distribution and retailing; and final consumption. The identified data in Table 3 will be referred to in each of the next three section to analyse the *Regie’s* operation within the supply chain in comparison with the endgames proposals.

**Table 3:** Summary of endgame approaches within the supply chain operational remit theme of the proposed MOEM

Sub-themes	Liberman [17]	Borland [8]	Callard [9]	Thomson et al. [18]	Thomson et al. [11]	Gray [19, 20]	Smith et al. [21]
MOEM function within the supply chain							
	The agency has an overarching regulatory presence on all the processes of the supply chain	Between the manufacturers and the distributors: take over the marketing component	• Transfer the supply chain work to a non-profit agency removing the tobacco industry from the whole market •Tobacco growers, stakeholders, distributors, and retailers could remain in the market but should change to other goals as the tobacco market will vanish	•Similar to Borland but also regulate the retailers via licensing •Take over the marketing component	•The agency will be introduced before the manufacture in the supply chain and will supply suppliers with tradable quota •SOTM can participate in the auction (to control potential auction rigging by suppliers)	Same as Borland Take over the marketing component	A government tobacco monopoly be introduced, and act as sole buyer. The retailer will be removed from the supply chain and be replaced by government operated retailers
Product design							
(1) Reveal ingredient/content (2) Labelling (3) Packaging (4) Determine the tobacco manufacturing process	(1)(2)(3)(4)	(1)	(2)(3- Design and manufacture their cigarettes in ways that reduce their attractiveness or addictiveness)	(1)(3)	(2)(3)	(1)(2-Generic) (3)	(1)(2)(3)
Purchase							
(1) Become the sole purchaser of tobacco products from the manufacturer (2) Adopt a regulatory role over the work of the manufacturer (licensing) (3) Set the amount of tobacco product/or brands/or tendered amount to be allowed into the market (4) Set the whole price	(1)(2)(3)	(1)(3)(4)	(3)	(1)(3)(4)	(3- Quota on manufactured and imported products)(4)	(1)	(1)(3)(4)
Promotion							
(1) Control tobacco product communication (what goes on the pack) (2) Move into unbranded products (3) Control what retailers can communicate with consumers	(1)(3)	(1)(2)(3)	(1)	(1)(2)	(2)	(1)(2)	(1)
Distribution							
(1) Bind the distributor to a license agreement – full control (2) Take over distribution (3) Keep the relationship between the manufacturer and distributor (4) Complimentary intervention to address potential fake shortages of supply	(1)	(1)	(2)	(1)	(3)(4)		(2- Into government owned retail shops by removing the retailing sector and retailers)
Retailing							
(1) Bind the retailer to a license agreement – full control (2) Takeover the retail (3) Indirect control with licensing (4) Set retail price (5) Anti-monopoly laws (6) Support retailers to switch to alternative markets through financial reward/incentivise to be involved in cessation programmes (7) Restrict store density and location (8) Set condition under which products could be available in the market (9) Set age of purchase (10) Limit selling hours (11) Set limit on purchases to control secondary illegal sale to minors	(1)(4)(8)	(3)(4)(5)(7)	(2)(6)	(1)(7)(8)	(4- Price would increase as supply decreases)(8)(9)	(3)(4)(6)(7)	(2- Restricting sale to government owned retail stores)(4)(6)(7)(8)(9)(10)(11)
Final consumption							
(1) Ensure that demand is met (2) Act to reduce demand (3) Shift demand to less harmful products (4) Communicate with consumers: awareness; cessation; price change (5) Allow growing tobacco for personal use	(1)(2)(3)(4-cessation information)	(1)(2)(3)	(1)(2)		(5)	(3)	(1)

### Product design

The endgame literature highlights the importance of introducing an MOEM that controls product design to develop less harmful and less addictive products as part of a public health mandate.

The *Regie* has no strong incentive to implement product design measures that would reduce tobacco-related harm where they generate profits. However, the *Regie* was successful in lobbying the Minister of Finance to override a decree issued by him in 2013 (while the Minister of Public Health) that banned e-cigarettes and heated tobacco products, and issue a new decree in 2015 that regulates their sale. In 2019, Lebanon moved from a country that banned e-cigarettes and heated tobacco product to a country that sells them in its market [43].

The *Regie* along with the TTC were successful in advocating the MOF to delay the implementation of textual health-warnings in 2013 [51, 83] and consistently lobbied the MOF to avoid signing the decree for pictorial warnings [84], even though a study conducted among Lebanese youth revealed the perceived effectiveness of pictorial in addressing the health and the negative economic consequences of smoking [52].

### Importation/production

The endgame literature suggests that having an MOEM as the sole purchaser of tobacco products from the tobacco industry would allow it to regulate the manufacturers. In addition, the MOEM would be able to prevent the growth of the tobacco market and regulate tobacco prices. The endgame literature does not suggest a means to regulate tobacco growing as part of the tobacco trade. Only Callard et al. [9] mention it at all, and seem to assume it will eventually vanish with the decrease in tobacco consumption.

The *Regie* is the sole entity in Lebanon able to purchase from and negotiate trade agreements with TTC. Lebanon is one among five countries in the world that devotes more than 1% (3.2%) of its agricultural territory to tobacco farming [46]. The *Regie* has created an exchange deal with TTC to safeguard the local tobacco leaf growing industry. TTC buy local tobacco leaf and in return the *Regie* imports manufactured tobacco products to sell in the Lebanese market, buys the Virginia tobacco leaf to manufacture domestic brand cigarettes [49], and in the past few years, locally manufactures some international brands (discussed below).

Internal industry documents indicate that the TTC have used the Lebanese market in the past as an entry point for legally and illegally exporting tobacco products into neighbouring countries, taking advantage of the Lebanese government’s unwillingness or inability to control smuggling in the absence of adequate policies [44, 55]. By the first quarter of 2019, an Oxford Economics report put the illicit tobacco trade in the Lebanese market at 28.1% [85]. However, it is worth noting that this data has been subject to criticism given that the report was funded by the tobacco industry [86], and it has been reported that the TTC consistently over-inflate estimates of illicit trade [87].

The *Regie* has trade agreement with the TTC but furthermore, it acts towards facilitating their work in Lebanon. To illustrate, in the past few years, the TTC faced complications in the Lebanese market [88] as the sale of the domestic brand Cedars overtook foreign brands [89]. Starting from an agreement with PMI in 2017 [90], and built-on with later agreements with JTI (2018) [91] and BAT (2019) [92], the Regie began to locally produce international brands for domestic consumption and potentially export to neighbouring countries. The suggested reasons for these agreements are to reduce the “deficit in the balance of external payments”, hence alleviate the cost of shipping and customs fees [92, 93], and provide job opportunities for local businesses involved is secondary processing [92]. This change lowered the retail prices for the foreign brands [54] in the Lebanese market and, hence, increased their affordability. By making tobacco products more affordable, the *Regie* is, in effect, obstructing tobacco control measures.

### Promotion

The endgame literature highlights the importance of regulating product promotion. This was mainly related to the power of the MOEM to decide on product design, and what needs to be communicated about the products by the retailers and wholesalers. Borland’s [8] proposal for an MOEM is to takeover the market entirely, which would eliminate any potential commercial incentive.

Turning to Lebanon, direct promotion of tobacco products, via advertising (such as written and audio-visual media) [94, 95] is less prominent [53]. What is problematic is indirect promotion and sponsorship such as CSR activities [94] by both the *Regie* and the TTC. The *Regie* has no marketing department but, in 2016, the *Regie* launched its Sustainable Development plan: "Development Vision for a Brighter Tomorrow”. Since then, it has been conducting CSR activities in line with its set priorities [96]. It has been undertaking activities such as running sports events [43, 97], sponsoring awareness activities around illegal recreational drugs, and financially supporting municipalities while promoting these CSR activities as a means to improve the life of their communities [98]. The *Regie* even publicly donated a total of US$2.6 million to the Lebanese Army in 2013 and 2021 [99–101], and donated US$1  millionM to the government to help address the COVID-19 crisis, which in both cases directly violated the provisions of Article 5.3 of the FCTC [102]. The TTC are likewise involved in indirect advertising in Lebanon. They provide convenience stores with LED illuminated tobacco stands and store branding [47], and are involved in sponsorship activities in co-ordination with the *Regie* [103]. They also promote their products by distributing free samples and accessories [53, 104], though the involvement of the *Regie* in this is currently unclear.

### Distribution and retailing

The endgame literature suggests having an MOEM that could regulate wholesale distribution and/or retailing, either partially or fully, or even takeover their work in the supply chain. It is also clearly mindful that industry could work toward creating fake shortages and manipulate the price of products in the market. The endgame literature suggests that MOEM could regulate sales in retail stores by controlling store density, location, and selling hours, as well as suggests means to control retailers via anti monopolies laws and support them to transition away from selling tobacco or involve them in cessation programmes.

The *Regie* plays the role in between the TTC and wholesale distributors, and supplies domestic brands to wholesalers. There are around 800 licensed wholesalers [105] that, in theory, are effectively controlled by the *Regie*, which has the right to suspend their licenses [106]. The retailers, although licensed by the *Regie,* are not directly controlled by it, and communication with them is through the wholesalers which makes the retailers largely dependent on the wholesalers for their tobacco supply. Tobacco products are sold in almost every type of shop (including big supermarket, convenience, and grocery shops) [107], and there is no store density control or control over selling hours, unlike in the models proposed in the endgame literature.

However, the *Regie* does have authority over retail prices, the weekly tobacco supply quota (to control the market), and sets the profit margins for both wholesalers and retailers [49, 89, 108]. In practice this control is quite limited as consumers have complained about retail price manipulation, and the *Regie* has stated that it is the role of the consumer protection unit within the Ministry of Economy and Trade to monitor the tobacco product price in the market and not the *Regie.*

The wholesalers seem to have formed a cartel, which controls most of the licenses, including those of other distributors who sign-over the control of the licence through a secret legal agreement. The large ones effectively control the wholesale market [109] and these have direct links with TTC [54, 109] where they can agree to create artificial shortages to increase their profit [109] or promote a particular brand over another, an action that at least one senior *Regie* employee confirmed being aware of [110]. Furthermore, they have the power to manipulate the retail price**.** In 2017 a tax increase on tobacco products was discussed by the government, and before any official decision was taken, the wholesalers increased their price for retailers without consulting with the *Regie*. It took the *Regie* four days to act (i.e., to increase its price to wholesalers). This delay by the *Regie* was suggested to have cost the *Regie,* and hence the Government treasury, LBP1.5bn (US$1million), which was instead captured by the wholesale distributors. It is not clear if the *Regie* lacks procedures and plans to deal with these wholesalers, or if it is simply disinclined to act due to corruption [109] or to at least safeguard the close relationships between politicians and distributors [54]. To our knowledge, the *Regie* had never suspended a license to a distributor until August 2021 when the COVID-19 pandemic and economic collapse changed the picture significantly [111]. In the absence of any sanctioning measures by the *Regie*, when price manipulation occurs, or a fake shortage is created, the retailers have to abide by the wholesalers’ decisions.

Nonetheless, some retailers also take advantage of retail price manipulation by charging prices above those set by the *Regie* to compensate for their low margin of profit (also set by the *Regie*). Through informal conversations with retailers, it was reported that illicit tobacco products were being sold alongside licit products. However, the illicit product is mainly sold outside Beirut, where there are fewer customs police.

The *Regie*, if it wants, and in line with the endgame literature on MOEM, can play a more regulator type of role towards controlling the wholesaler-retailer component of the supply chain. In 2021 during the COVID-19 lockdown, it took a one-time decision, that was described as an 'unprecedented measure', to bypass the wholesale distributors and to directly provide the retailers with tobacco products. The decision was perceived as an action to curb the monopolisation of the market by the wholesale distributors [112].

### Final consumption

The endgame literature is supply-side oriented, although protecting consumers from the harm of tobacco is their ultimate target. The proposals suggest that the MOEM should adopt an incremental approach to shifting consumers towards alternative products that are less addictive in order to avoid the further development of a black market and an increase in smuggling. In addition, they raise concerns that countries with relatively permeable borders could face smuggling if they implemented an MOEM [8, 11].

Contrary to the endgame proposals, the *Regie*’s approach to ‘increase consumer satisfaction’ is to make tobacco products available and affordable in the Lebanese market. The aim of the *Regie* is far from shifting consumers from the harm of smoking (although it has advocated for the introduction of e-cigarettes in the Lebanese market). In its sustainability report of 2016, preventing youth smoking is only the 8^th^ out of 15 of its sustainability drivers. Preceding it are, in order: ‘*combating illicit trade; sustainable agriculture; fighting child labour, consistency of product, development of employees’ skills reduce of energy consumption, consumer satisfaction’.* Furthermore, it was in fact removed from the 2017 report which only featured 14 sustainability drivers [96, 113].

Unsurprisingly, Lebanon has some of the highest global rates of tobacco use compared to other middle-income countries [114] and, according to some studies, these are only increasing. For example, in September 2019 it was reported that 34% of men, and 21.2% of women are daily cigarette smokers, and 26.5% and 24.3% are respectively, daily waterpipe smokers [114]. A different study in May 2020 suggested 48.6% of adult men and 21.5% of adult women are cigarette smokers [53]. These high rates of tobacco use are associated with an annual cost of smoking to the Lebanese population of approximately US$326.7 million, equivalent to 1.1% of Lebanese GDP [49].

## Discussion

Our paper identified seven endgame proposals featuring MOEM which were mapped against two main themes of structural governance and operational remit. Our findings identified that the endgame proposals underlined the importance of a congruous governance structure for the suggested MOEM as well as the significance of regulating the different segments of the supply chain to address the TTC influence on the supply side. The analysis of the *Regie* relative to those proposals revealed that the endgame literature did not fully appreciate the extent to which any MOEM could end up acting similar to a TTC. This is a danger emphasised in Article 5.3 of the FCTC, which calls for SOTM to be treated as the tobacco industry. Indeed, the analysis showed how the *Regie* acts like a TTC in many ways, affecting both its governance and operational remit. Moreover, the endgame literature has only vaguely addressed the implications of political context when establishing an MOEM [8, 11, 21, 115]. The case of the *Regie* has demonstrated how the MOF and other political elites have been actively involved in obstructing tobacco control measures, and hence that the endgame literature ignores a much-needed requirement for strong political leadership.

Thus, the first pre-condition for the MOEM to be a success, and for Lebanon to advance tobacco control, is to have strong political leadership and support that could enhance the context for policy change [115]. The second pre-condition for the MOEM is to address how to regulate tobacco growing, as farming is currently neglected by the endgame literature although it is it is an important element of the supply chain and could be part of the supply chain for several countries that would eventually adopt MOEM.

In the following discussion we draw out lessons for both MOEM and for Lebanon. Although certain elements of the *Regie* are context specific, it still holds some valuable lessons for a proposed MOEM.

### Lessons learnt for MOEM

Overall, the endgame proposals highlight the crucial significance of having an MOEM with a public health mandate, and preferably also a not-for-profit one too. The case of the *Regie* illustrates how difficult it would be for an already existing SOTM to adopt a public health mandate given its profit-making objective [22, 116–119]. The financial issues caused by the COVID-19 pandemic for governments around the world will only have increased the difficulty of shifting to a non-profit making model [120].

Hence, focusing on public health and dropping profit might seem more wishful than practical at this stage. Consequently, to ensure a more plausible approach, endgame proposals need to ensure two specific elements/goals are accounted for when considering an MOEM: an appropriate governance structure and a carefully designed system of financial incentives.

### Ensure an appropriate Governance structure

In their narratives, the proposals require the MOEM to be independent from political interference and impose regulatory measures to control the operation of the supply chain and the work of the different stakeholders (manufacturers, distributors, and retailers). The endgame literature also emphasises the importance of monitoring the work of the MOEM. However, the case of the *Regie* has demonstrated that an appropriate governance structure is challenging within a prevailing SOTM. This is further challenged by the lack of clear accountability mechanisms and a functional regulatory body, which combine to preclude holding the *Regie*, TTC, and politicians accountable. We note that the endgame proposals did not outline how they could ensure a functional regulatory body; or include clear accountability mechanisms; or how to deal with an MOEM if it begins it behaves similarly to tobacco industry.

Accordingly, for endgame proposals to ensure an appropriate governance structure of the MOEM they need not only to meet their suggested features and characteristics, as summarised in Table 2, but also ensure other actions are met beyond the features in Table 2. Therefore, there is a need to have established a functional independent regulatory agency that works: (i) to create a suitable accountability mechanism for the MOEM; (ii) to ensure when implementing the provision of Article 5.3 of the FCTC [121], they acknowledge the possibility that the MOEM could act similarly as tobacco industry and hence need to be treated as such.

### Adjusted financial incentives

The endgame proposals highlight the need to address financial interests to avoid maintaining tobacco sales and acknowledge the need to prevent financial gain. Some proposals suggest earmarking any profit for funding tobacco research, awareness campaigns, and quitting initiatives. The case of the *Regie* has demonstrated this issue by showing how it acts similarly to the TTC by seeking financial gain. Therefore, the MOEM should: (i) recognise that tobacco taxes can still go to government irrespective of which type of organisation produces tobacco products, and hence only profit related dividends paid to government would need to be avoided; (ii) remain distant and independent of government by limiting its role to providing information to enable taxation, rather than being involved in decision making on taxation.

### Further consideration for the endgame literature: Tobacco growing

The investigation of the *Regie* revealed two issues related to tobacco growing that are of significance to discussions around MOEMs. Firstly, the *Regie* tends to portray tobacco control as negatively affecting tobacco farming, farmers, and hence supply chain economics, using such arguments as tools to weaken tobacco control in Lebanon while reinforcing the position of TTC [54]. Secondly, the *Regie* keeps tobacco farmers dependent on tobacco planting by portraying it as a secure source of income [27, 54, 122] and reinforcing this through subsidies. However, unlike the *Regie*’s claims, tobacco farmers appear willing to shift to other forms of farming if opportunities arise [27, 54], which could allow them to become independent of the politicians, as they would no longer need licensing to execute their work. It is mainly the politicians that control tobacco license distribution and tend to give it to their allies [123].

Hence, any proposal advocating for regulating tobacco growing as part of the operational remit of the suggested MOEM, needs to be aware that, in most cases, totally removing tobacco farming from the supply chain would be extremely difficult. Instead, it needs to be gradually reduced in line with reduction in tobacco use. In other words, farmers can and should be transitioned out of leaf as part of wider tobacco control efforts. Until such a transition can be completed, tobacco farming should be regulated by the same regulatory body (as mentioned above) that regulates the MOEM to avoid adversely impacting tobacco farmer livelihoods. Such regulation might include ensuring tobacco farmers are protected from political interference while also providing them with access to financial support, technical skills training, or markets to sell alternative crops. Such initiatives could be funded by hypothecating profit from the MOEM.

### Lessons learnt for Lebanon

Our investigation of the *Regie* confirmed that endgame strategies are far from the agenda of the Lebanese government. This is mainly related to the lack of priority for public health over financial gain. Nonetheless, within the exploration of the *Regie* and the endgame strategies for MOEM, there are lessons to be learnt for Lebanon to help it move towards reducing tobacco use consistent with the endgame strategies.

**Fig. 2:**
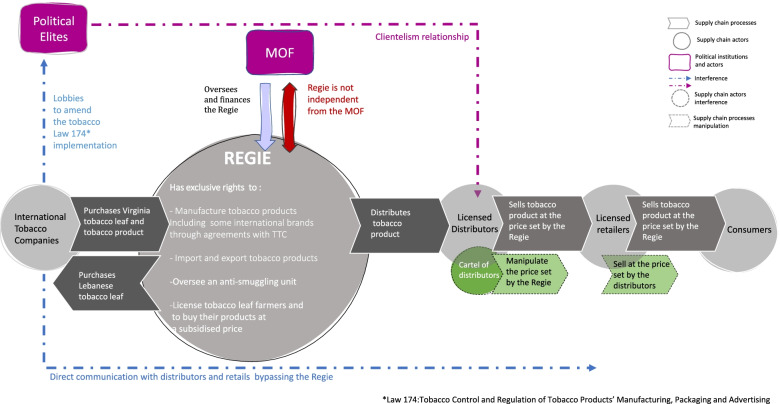
The case of the *Reg*ie: element of its tobacco supply chain in real-world setting. Source: Authors’ interpretation drawing upon [28, 29, 31]

Unlike the simple supply-chain structure illustrated in Fig. 1, the real-world situation of the governance structure and the operational remit of the *Regie* is much more complex. This is because, in practice, the Regie lacks independence from the government (MOF), the political elites maintain a clientelism relationship with the wholesalers, and the TTCs lobby politicians while bypassing the Regie to establish direct communication with the wholesalers and the retailers. Additionally, a cartel of distributors manipulates the retail price set by the *Regie*. Figure 2 illustrates these complexities.

Accordingly, in the case of Lebanon, there is a need to address how to ensure an appropriate governance structure and adjust financial incentives.

### Ensure proper Governance structure

The main obstacle towards ensuring an appropriate governance structure in the case of the *Regie* is its political affiliation with, and the interference of, politicians in its work to serve their vested intertest. Therefore, one-way forward is to adapt measures that would break the *Regie’s* political relationships. The route to achieve this could be by privatising the *Regie*. However, such an approach is unrealistic at the moment, and would in any case be difficult within the Lebanese sectarian based political system. In addition, the *Regie*, supported by the TTC, has always opposed such an approach [124]. A recent study [122] also concluded that privatisation would be unlikely and even undesirable given the high level of corruption, lack of sound regulatory measures and transparency that could lead to rapid gain of revenue by the politicians and their partners.

Another route would be to sustain the *Regi*e in its current form but enhance the regulatory role of the NTCP [65]. This seems much more plausible, although to achieve it, the NTCP would have to: (i) put in place accountability mechanisms to hold the *Regie* accountable for its decisions and to prevent the *Regie* from jeopardising the public health of the Lebanese population in its drive for profit; (ii) draft guidelines (in line with the provision of Article 5.3 [121]) to prevent government officials from communication with the tobacco industry and any possible collaboration with them [43] and; (iii) enhance its employees professionalism and independence by ensuring proper pay and benefits.

### Adjusted financial incentives

Given the Lebanese context of weak governance, economic instability, and endemic corruption [45, 51, 53, 125], not to mention the fact that the *Regie* generates significant income for the state [122], regulating financial incentives is complex. Hence, one way forward would be to address the dependance of the Lebanese government on the *Regie’s* revenue. To achieve this there is a need to earmark profits away from government. The MOF would still collect money from tobacco taxes (but not from profit dividends). If this were implemented, it would de-incentivise it from growing its market and potentially it could therefore restrict the work of the *Regie.*

Furthermore, another means to control financial incentives from the sale of tobacco products could see the *Regie* taking over the role of the distributor in the supply chain, allowing it to directly communicate with retailers, thereby preventing price manipulation. By doing so it could further restrict outlet locations and numbers, and directly control retail prices as suggested by the endgame literature. Or, in the instance the distributors are kept in the market, the *Regie* could exert meaningful control over their role though enforcing strict regulation such as stopping their license to break the existing cartel and the vested interests between the distributors, TTC, and politicians.

## Conclusion

The implementation of tobacco endgame strategies of any type is now closer than ever. New Zealand is currently considering an action plan based on a wider endgame strategy (i.e. non-MOEM) to achieve its ‘Smokefree 2025 goal’ [126, 127]. This paper examined the features of endgame proposals that would introduce some form of monopoly in order to understand what the suggested MOEM is expected to achieve and to explore the extent to which the various features already exist in the case of the *Regie,* a pre-existing SOTM in Lebanon.

Using the *Regie* as a practical example allows us to effectively revisit both the potential and the pitfalls of endgame strategies intending to adopt MOEM or intending to reform a pre-existing SOTM to reduce tobacco use in line with endgame models. Specifically, the *Regie* as an SOTM could encourage similar countries with an SOTM (particularly those in an LMIC context), and also those with state-owned oligopoly tobacco firms, to consider the suggested lessons and potentially start thinking of the possibility of setting endgame goals. However, countries using the lessons learnt from the *Regie* should account for its specific context and hence adjust accordingly.

The main limitation of this paper is with respect to the data. Given the scarcity of academic literature related to the *Regie*, much of the data was collected from the *Regie* website and local media reports. These data sources might have not been entirely independent and free from bias. However, data triangulation was undertaken whenever possible, and we are confident that our results are broadly reliable. Furthermore, we recognise that we were not able to undertake a multisectoral approach [128], which involves considering multiple sectors (e.g. economy, environment, health), which could have generated additional insights given the cross cutting nature of tobacco in terms of advancing an endgame. Future research might productively undertake such a multisectoral approach to investigate the impact of the wider dynamic of an MOEM approach to a tobacco endgame.

The recommendation for countries considering an MOEM and those for Lebanon are aligned, with the key difference being in the implementation. Possibly in the future, there will be a need to draw further lessons from countries that have already implemented endgame strategies with an MOEM, and hence the need of further studies in this area.

Our choice of the *Regie* was influenced by our knowledge of the Lebanese tobacco control context and we are aware that Lebanon is far from implementing endgame strategies. Nevertheless, by exploring the *Regie*, we are contributing to the scarce literature on SOTM, especially as we are the first to consider this organisation in this context as far as we are aware [22]. In addition, we are also contributing to the knowledge of endgame strategies by providing lesson learnt for the possible use of MOEM, thereby enhancing their practicality for real-world use.

## Data Availability

All data generated or analysed during this study are included in this published article.

## Abbreviations

FCTC: Framework Convention on Tobacco Control
GTIII: Global Tobacco Industry Interference Index
MOEM: Monopoly-oriented endgame model
MOF: Ministry of Finance
MPs: Member of parliament
NTCP: National Tobacco Control Programme
SOTM: State-owned tobacco monopoly
TTC: Transnational Tobacco Companies
